# Next viewer–development of a software for the spatial computing data glasses apple vision pro for patient-specific preoperative planning and navigation

**DOI:** 10.3389/fdgth.2026.1764807

**Published:** 2026-08-28

**Authors:** Conrad Günther, Dirk Winkler, Svenja Jung, Martin Hoffmann, Katharina Scheidt, Fabian Kropla, Erdem Güresir, Ronny Grunert

**Affiliations:** 1 Department of Neurosurgery, University Hospital Leipzig, Leipzig, Germany; 2 Fraunhofer Institute for Machine Tools and Forming Technology, Zittau, Germany

**Keywords:** computer assisted surgery, mixed reality, neurosurgery, preoperative planning, spatial computing

## Abstract

**Objective:**

This study aims to evaluate the clinical utility of *nextViewer*, a novel spatial computing application for the Apple Vision Pro, to determine its efficacy in enhancing patient-specific neurosurgical preoperative planning, spatial comprehension, and multi-user team communication.

**Methods:**

A complete translational workflow was established, encompassing the segmentation of high-resolution CT/MRI scans into patient-specific 3D models and their integration into the spatial computing environment. The software leverages integrated eye- and hand-tracking to enable controller-free interaction, including 3D model manipulation, surgical trajectory drawing, and multiplanar finger-navigation. A systematic clinical evaluation was conducted with 15 experienced neurosurgeons. User satisfaction regarding user-friendliness, surgical planning benefits, system stability, and visualization quality was assessed utilizing a Likert-scale questionnaire ranging from −2 (strongly disagree) to +2 (strongly agree).

**Results:**

The application successfully facilitated intuitive, real-time manipulation of complex anatomical models, utilizing transparencies to visualize underlying critical structures. The finger-navigation feature—providing immediate crosshair updates in corresponding coronal, sagittal, and axial planes—was identified as a unique selling point for spatial orientation. Furthermore, the collaboration mode enabled synchronized, multi-user case discussions. The systematic evaluation yielded a highly positive total score of +1.7. Surgeons specifically highlighted the controller-free interaction and the exceptional graphical resolution as significant advantages over legacy visualization systems.

**Conclusions:**

Controller-free spatial computing via the Apple Vision Pro significantly enhances neurosurgical preoperative planning. By improving real-time spatial awareness and enabling seamless interactive simulations, the nextViewer app bridges the gap between theoretical augmented reality concepts and practical clinical application. The system demonstrates strong potential to become a standard tool in neurosurgery, establishing a robust foundation for future integration with telemedicine and advanced surgical navigation.

## Introduction

Neurosurgery demands the highest level of precision due to the complexity and sensitivity of neural structures, making meticulous preoperative planning essential for successful surgical outcomes. In recent years, extended reality technologies—encompassing Virtual Reality (VR), Augmented Reality (AR), Mixed Reality (MR), and Spatial Computing (SC)—have fundamentally transformed this planning process. These modalities enable surgeons to visualize patient-specific anatomy in unprecedented detail and in an intuitive, interactive manner (1, 2).

### Virtual reality

VR enables surgeons to interact with patient-specific 3D models—reconstructed from CT, MRI, or angiography data—within a completely immersive virtual environment. This immersion significantly improves spatial understanding in complex cases, such as tumors or vascular malformations (1, 3). Anthony et al. (3) demonstrated that utilizing 360° VR models for planning intracranial tumor resections greatly enhances the comprehension of anatomical relationships and surgical access routes (3). Furthermore, Colombo et al. (4) found that VR applied to aneurysm surgery can contribute to shorter operative times and superior anatomical understanding (4). Commercial VR systems (e.g., Surgical Theater or Brainlab VR Viewer) already offer functionalities for resection simulation, vessel visualization, and virtual surgical preparation (5), while also serving as an excellent tool for patient communication and informed consent (6).

### Augmented reality

While VR immerses the user completely, AR bridges the gap between preoperative planning and intraoperative execution by projecting supplementary 3D data—such as the position of a blood vessel or tumor—directly into the surgeon's real-world field of view (7). AR improves the visualization of complex anatomical structures prior to and during surgery (39, 40), such as spatial orientation for vessel courses in liver surgeries (8), and is similarly utilized in neurosurgery to visualize tumors and critical structures (9).

Specifically, AR has been successfully applied to the planning of craniotomies (10, 42), the visualization of tumor margins and neurovascular structures (11, 12), and the accurate placement of pedicle screws (13, 41, 43, 44). Ivan et al. (10) demonstrated in a pilot study that AR-supported craniotomy planning can reduce the size of skin incisions and bone openings while better sparing critical structures (10). In vascular neurosurgery, AR supports the treatment of cerebral aneurysms and stent selection, achieving a localization accuracy of 2–3 mm (14, 15) Furthermore, integrating AR with neuronavigation systems holds the potential to improve real-time intraoperative guidance and reduce the cognitive load on operators (2). However, traditional AR systems often lack the capability for direct, intuitive interaction with the overlaid virtual models.

However, traditional AR and VR systems present significant limitations for seamless clinical integration. VR completely isolates the surgeon from the clinical environment, while many AR systems rely on cumbersome external physical controllers or lack the capability for direct, intuitive, real-time interaction with the overlaid virtual models. Furthermore, synchronous multi-user collaboration around a shared holographic model remains a technical challenge.

### Mixed reality and spatial computing

Mixed Reality and Spatial Computing address these interaction limitations by enabling surgeons to actively manipulate and move holograms anchored as virtual objects within the real space (16). MR is increasingly utilized for surgical training, simulation, and navigation-supported intraoperative assistance (17–21). In cranial neurosurgery, MR aids in tumor resections and aneurysm planning by providing shared visual platforms for surgical teams, while also mitigating preoperative anxiety through enhanced patient education (4, 11, 22, 23).

The recent introduction of the Apple Vision Pro (Apple Inc., Cupertino, USA) in 2024 marks a significant milestone in Spatial Computing. Featuring high-resolution displays (23 million pixels) and highly precise, integrated eye- and hand-tracking, the device presents an attractive platform for medical applications (24, 25). Early reports indicate its strong potential for surgical planning of the skull and spine, as well as for medical education and intraoperative support (26, 27).

Looking beyond standalone spatial computing, Weyhe et al. found that integrating AR and MR into robot-assisted systems (e.g., the DaVinci system) can further increase precision (28). Supported by AI, such platforms could enable autonomous interventions in the future (29).

### Objective

**Figure 1:**
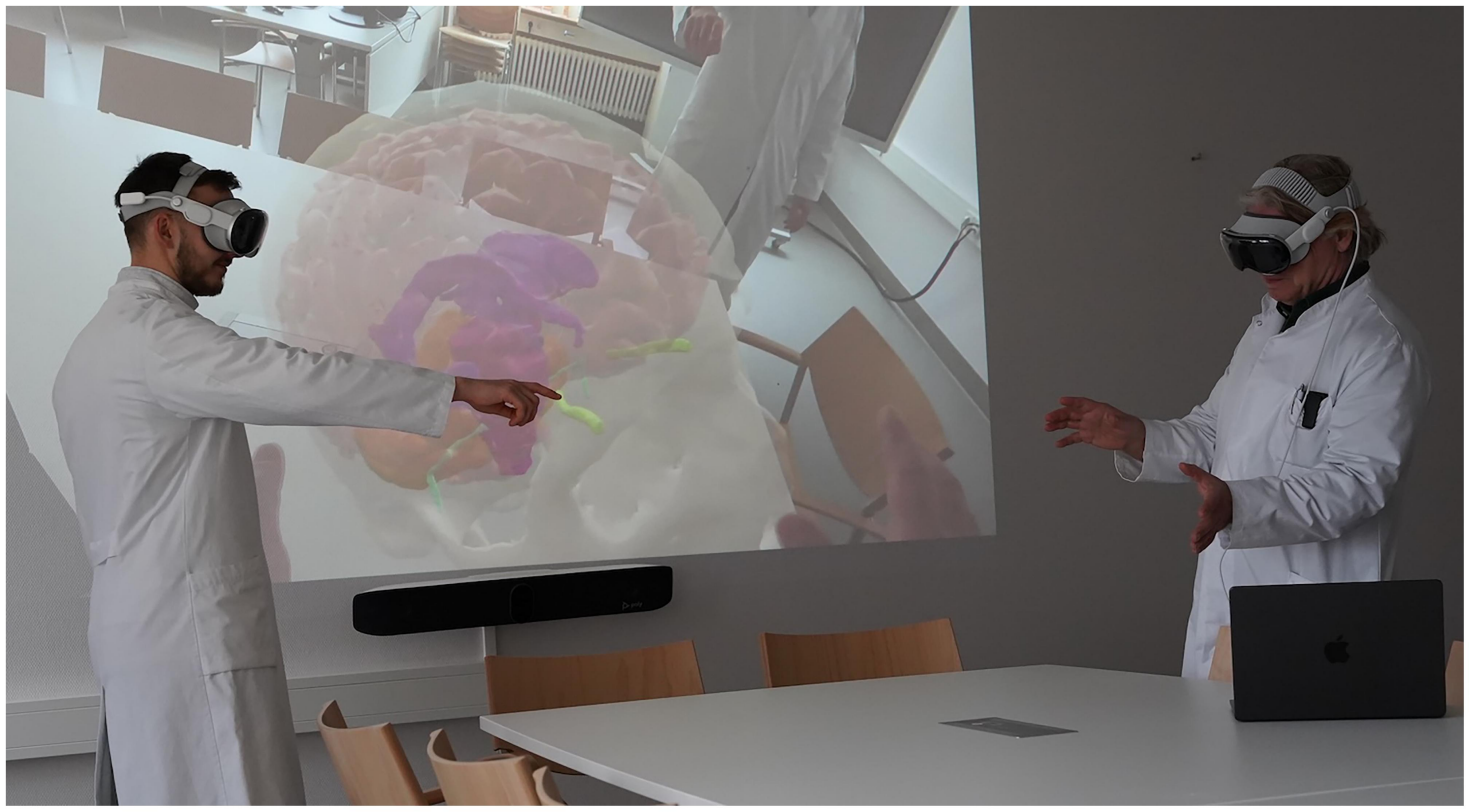
Application of the spatial computing glasses apple vision Pro with the developed app to discuss patient-specific surgical access planning during the morning neurosurgical meetings.

Despite these technological advancements, comprehensive clinical evaluations of multi-user planning and real-time spatial navigation capabilities remain scarce. Therefore, the objective of this study is to evaluate the clinical utility of *nextViewer*, a novel spatial computing application developed for the Apple Vision Pro. Rather than merely presenting a technical proof-of-concept, this work investigates the system's efficacy in enhancing patient-specific neurosurgical planning. We hypothesize that the application's unique combination of controller-free 3D model interaction, marking functionalities, and multiplanar finger-navigation will significantly improve spatial comprehension and team communication (Figure 1).

## Methods

**Figure 2:**
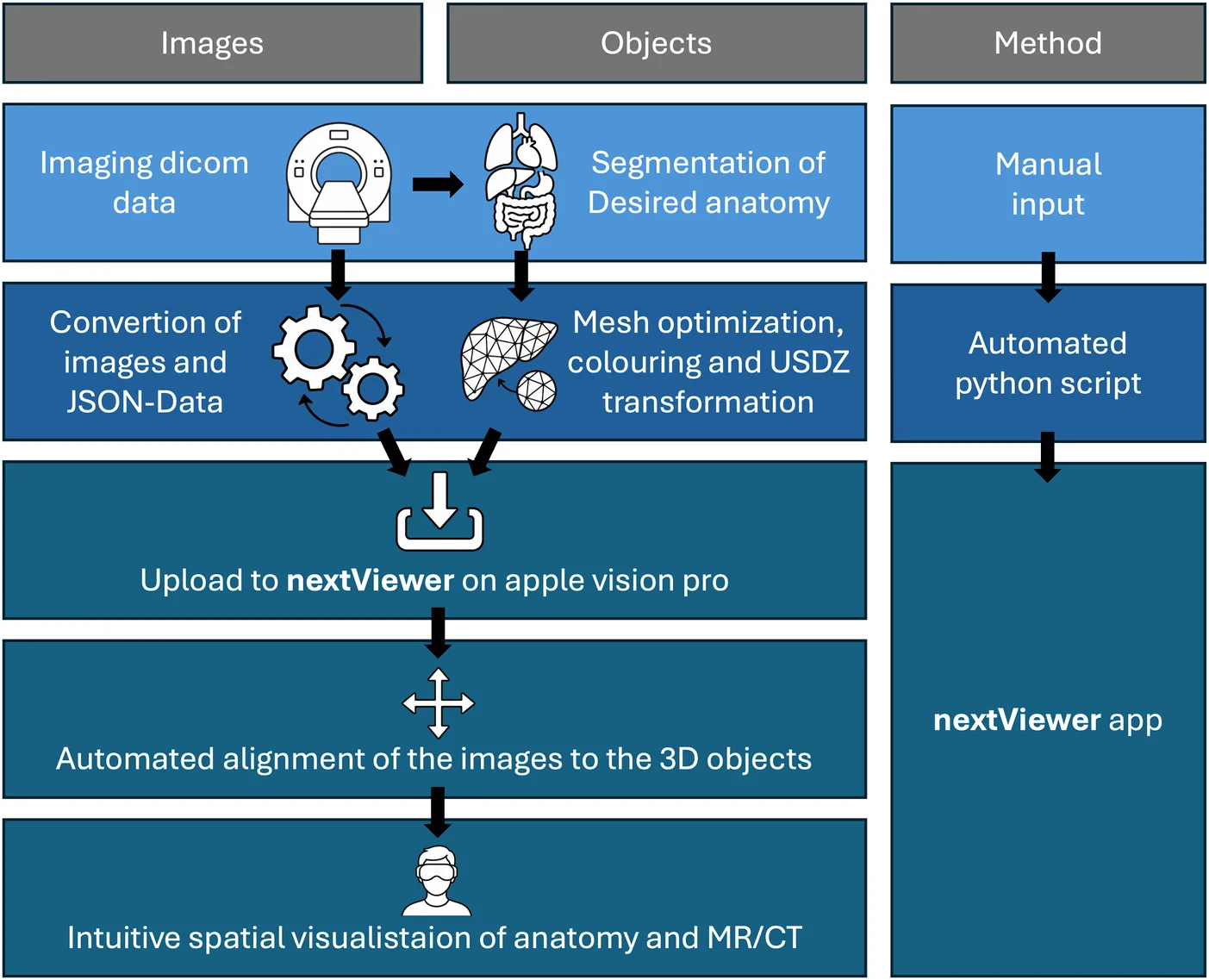
Workflow of the required tasks from medical image acquisition until export and use with the vision Pro.

For the Apple Vision Pro spatial computing data glasses (Apple Inc., Cupertino, USA), a software was developed in the Apple programming environment Xcode using the Swift programming language, which allows interaction with patient-specific virtual 3D models. The following process chain is required to create virtual anatomical patient data and import it into the Vision Pro (Figure 2).

### Medical image acquisition

A high-resolution CT or MRI scan of the patient is created, ideally with a slice distance of 1 mm. The image data is exported in DICOM format being the standard format for medical images.

### Image conversion

The medical image data is required for surgical navigation. Since the developed software on the Vision Pro cannot process native DICOM files, a conversion to the JSON format is required. For this purpose, an automated Python script was implemented so that the user can perform the conversion from DICOM to the JSON format with a single click.

### Segmentation

The relevant anatomical structures are marked using segmentation software. In our case, we used the open-source segmentation software 3D Slicer. The anatomical structures can be colored as desired and also given transparency.

The reconstructed virtual 3D models are then exported as OBJ files, which also contain the color information.

### Postprocessing of the virtual 3D-models and conversion into the USDZ format

Since the app developed for Apple Vision Pro utilizes the Apple-native USDZ format for 3D objects, a conversion from the OBJ format is necessary. An automatic software routine was developed for this purpose allowing all 3D objects to be converted with just one click.

### Importing of the 3D-data into the vision pro

After the virtual anatomical 3D models have been converted to the USDZ format, the data is imported into Apple Vision Pro via iCloud. In the next step, this data can be used with the developed app nextViewer.

### System architecture and app features

The Apple Vision Pro was specifically chosen over legacy mixed-reality headsets (such as the Microsoft HoloLens 2 or Oculus) due to its superior graphical resolution and its controller-free ecosystem. The *nextViewer* app was developed in the Apple programming environment Xcode using the Swift programming language. The app is navigated using the eyes (functioning as a cursor) and controlled by bringing the thumb and index finger together (functioning as a click). The features available to the surgeons are subdivided into three primary sections: Viewing, Drawing, and Navigation.

### Viewing

**Figure 3:**
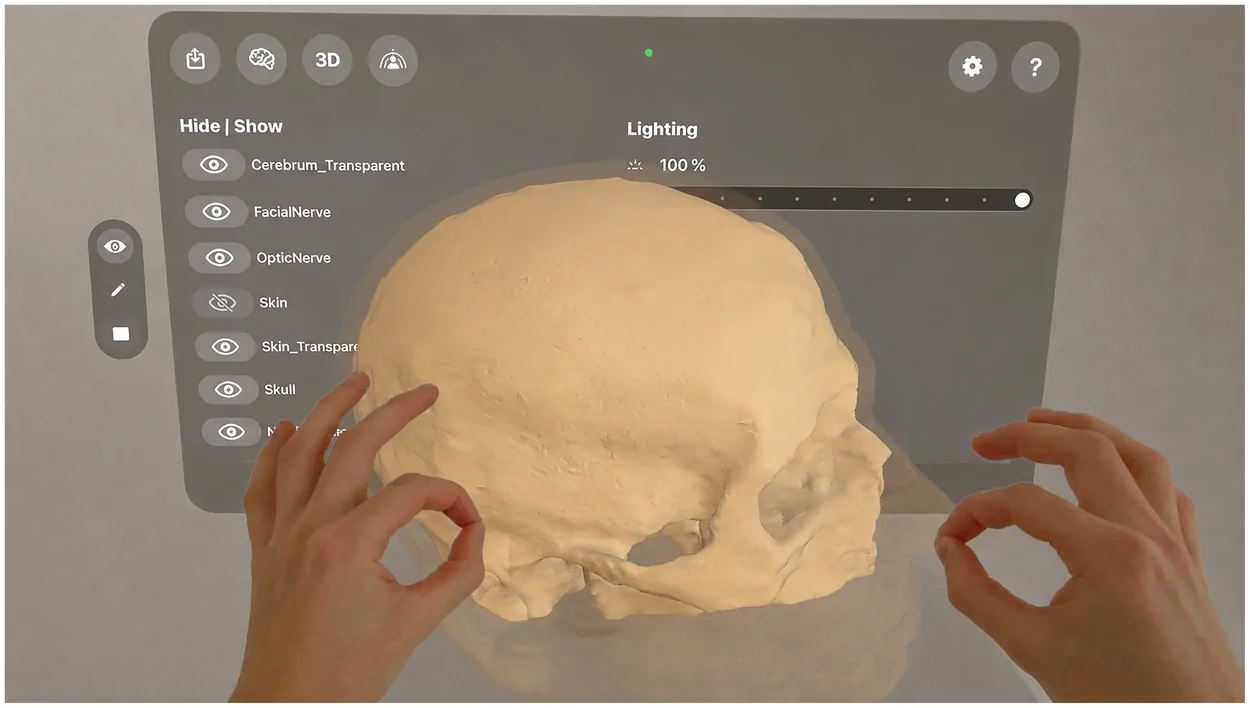
Interaction with the patient-specific 3D model using the eyes and hand gestures to move and scale the model as well as to show and hide single anatomical structures.

The patient-specific 3D model can be moved and rotated freely in space (Figure 3). Fine-grained movement is possible using sliders, and zooming can be performed using a gesture with both hands. Additionally, all anatomical structures are listed on the left side of the menu and can be individually shown or hidden by selecting the eye icon.

### Drawing

**Figure 4:**
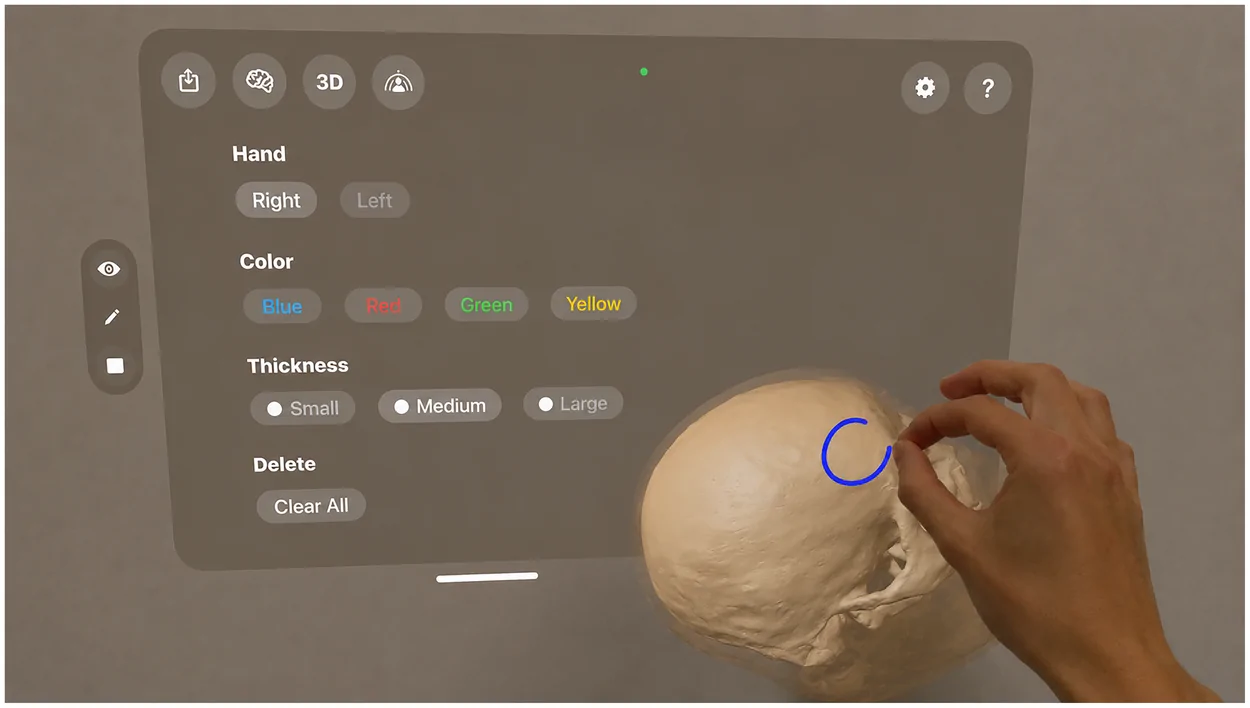
The software enables the surgeons to draw surgical access routes and trajectories.

This feature allows users to draw lines in space using finger gestures to visualize surgical access routes or trajectories. It is possible to color the lines differently and vary their thickness. A virtual eraser can be utilized with a hand gesture to delete sections of the drawn lines or remove them completely (Figure 4).

### Navigation

**Figure 5:**
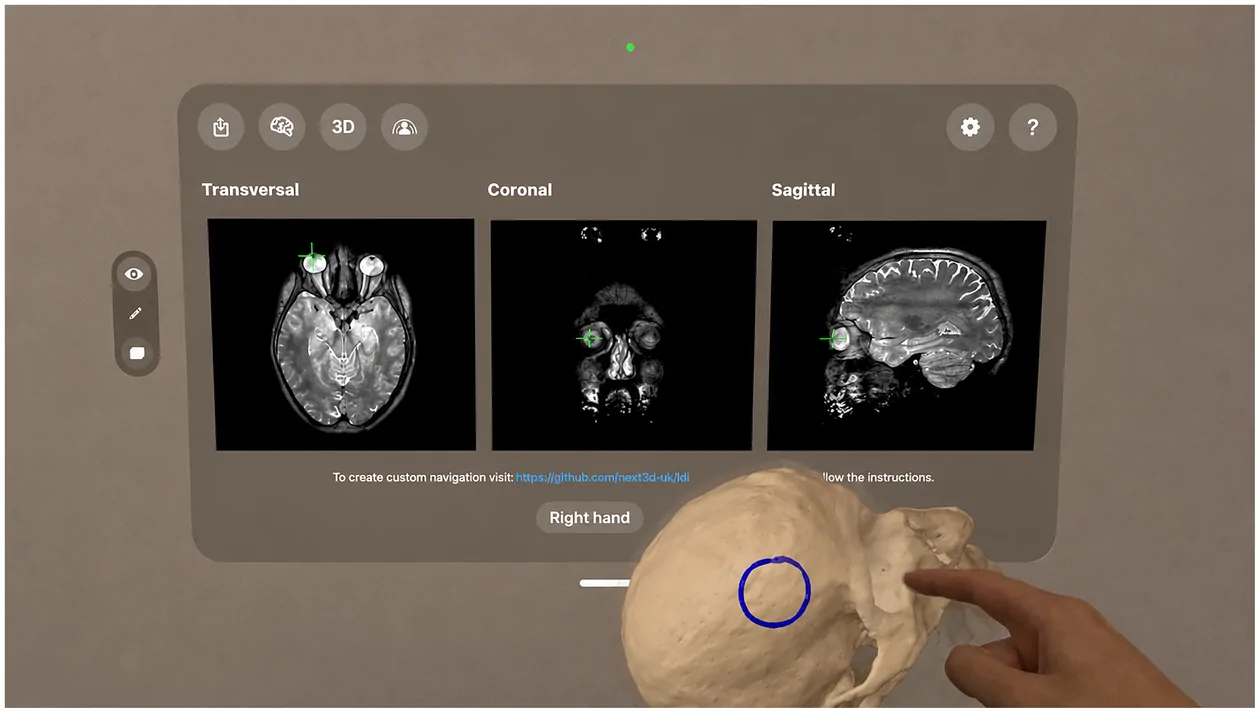
With the help of the navigation tool the surgeons can see inside the corresponding CT/MRI images the current position of the fingertip in the anatomical 3D model.

**Figure 6:**
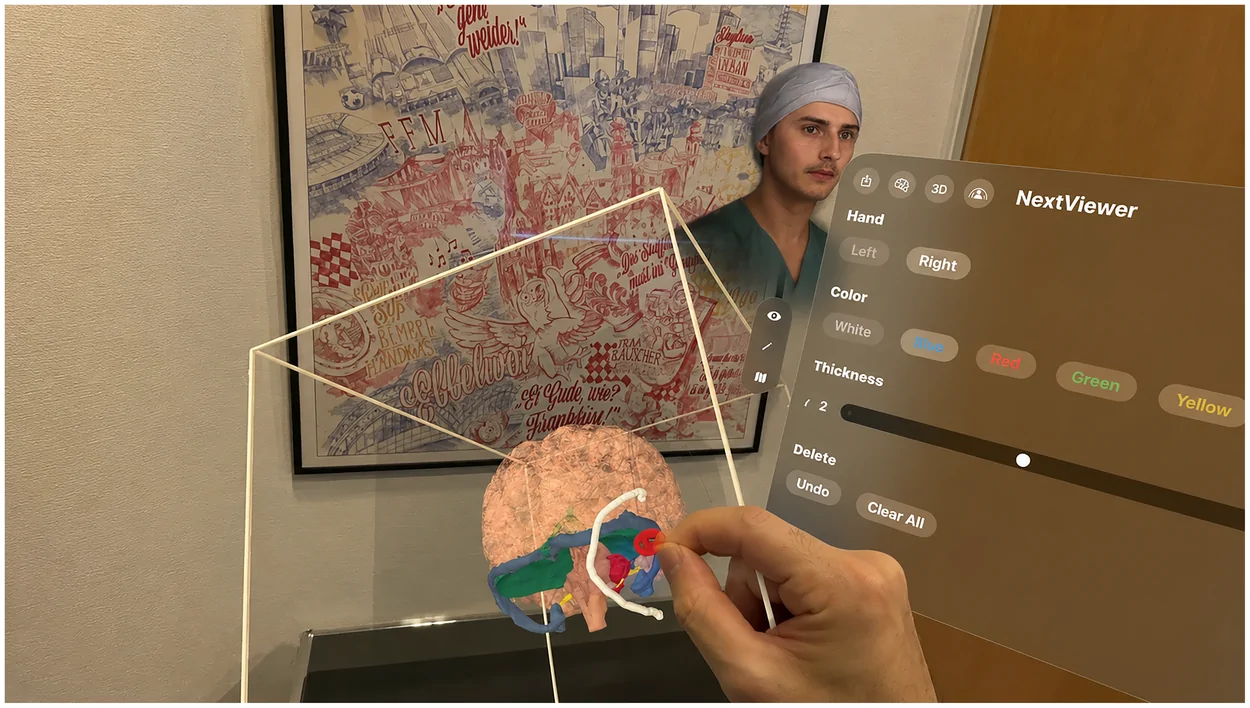
The collaboration feature can be used to discuss the surgical strategy and planning with several surgeons in one room or different locations.

The *nextViewer* app enables surgeons to perform real-time surgical navigation within the 3D model using their index finger. When this function is activated, the coronal, sagittal, and axial section planes from the CT or MRI are displayed. The position of the fingertip is displayed in real time as a crosshair in the corresponding section planes, automatically updating as the finger moves (Figure 5). Additionally, a Collaboration Mode was integrated via SharePlay, ensuring that the view of the 3D model is permanently synchronized across multiple users. This allows several surgeons to discuss patient-specific strategies simultaneously, either in the same room or remotely via a FaceTime call (Figure 6).

### Study design and evaluation of the app

A systematic clinical evaluation was conducted at the University Hospital Leipzig. The participant cohort consisted of 15 experienced neurosurgeons (each with >5 years of clinical experience in cranial and spinal procedures). The evaluations were integrated into routine clinical morning meetings. Participants were given a brief introduction to the controller-free interface and were then asked to perform a series of standardized tasks: loading a patient-specific model, utilizing the transparency tool to identify underlying structures, drawing a surgical trajectory, and navigating the multiplanar cross-sections using the index finger navigation. Following the tasks, user satisfaction was assessed. While standardized usability metrics such as the System Usability Scale (SUS) or UMUX are common for general software evaluation, we opted for a targeted Likert-scale questionnaire ranging from −2 (strongly disagree) to +2 (strongly agree). This custom approach was deliberately chosen to assess the *specific clinical utility* of the application—such as the improvement of spatial understanding of neurovascular structures and the accuracy of multiplanar crosshairs—rather than generic software usability. The evaluation criteria focused on four domains: user-friendliness, surgical planning benefits, system stability, and the quality of graphical visualization. To ensure full transparency, the complete questionnaire is provided as Supplementary Material 1. The overall aggregate score of +1.7 was calculated by computing the arithmetic mean of all responses across the entire questionnaire for all 15 participants.

## Results

A software has been developed for the Apple Vision Pro spatial computing glasses that allows surgeons to interact with patient-specific 3D models and use them to plan surgeries. The software makes it possible to move the virtual 3D models around the room as desired and also to project them onto the patient. By showing and hiding the various anatomical structures, surgeons can gain a better overview and focus on what is essential. The use of transparencies makes it possible to see through a structure in order to better recognize the areas behind it.

The marking option allows surgical access routes or resection lines to be marked and displayed in different colors and line thicknesses.

Finger navigation can be used to move through the virtual 3D model with the index finger and orient oneself within it. When the finger was moved, the displayed position was updated in real time with a crosshair in the coronal, sagittal and axial CT/MRI cross-sectional images.

With the help of the collaboration feature, multiple surgeons can discuss patient-specific planning in the same room or from different locations around the world.

All 15 participating neurosurgeons successfully completed the standardized tasks and the subsequent evaluation. The assessment focused on four primary domains to determine the system's clinical utility and technical maturity.

### Quantitative findings

**Figure 7:**
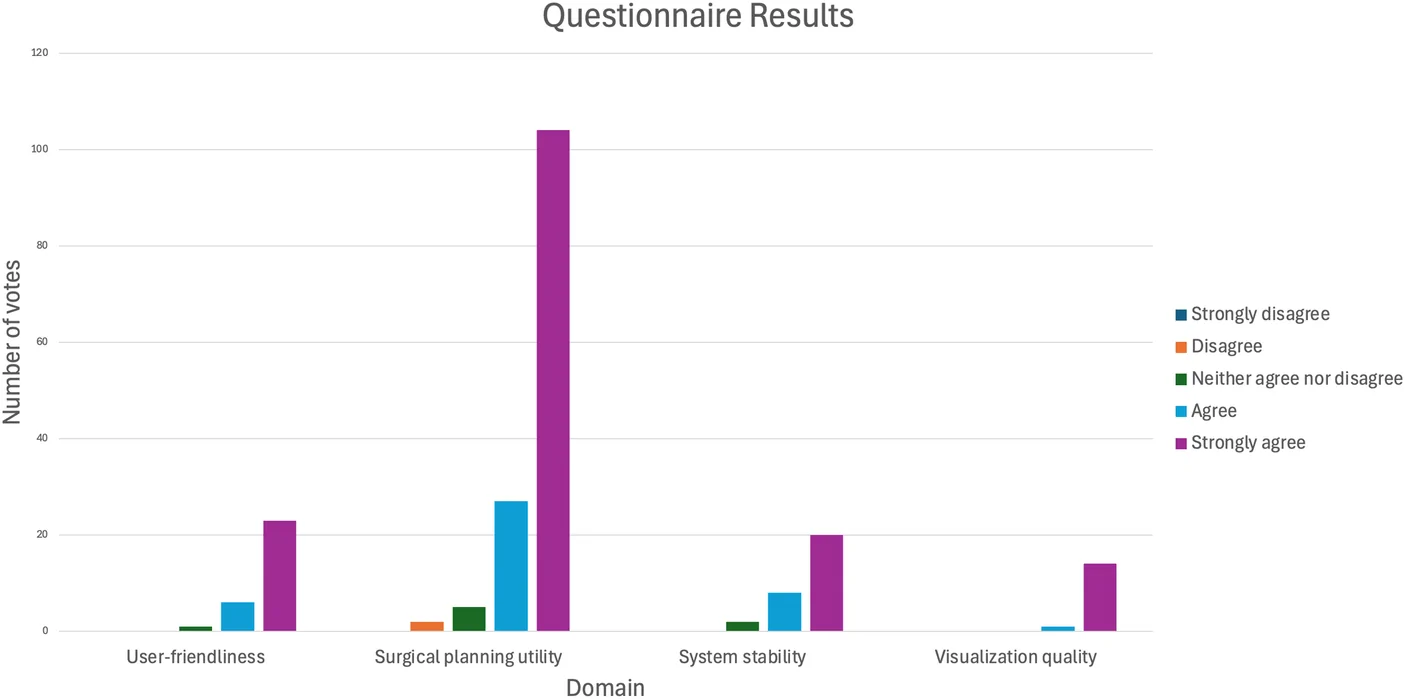
Bar chart summarizing Likert-scale evaluation scores across the four primary domains.

The overall aggregate performance yielded a highly positive total score of +1.7 (SD = 0.4) on the Likert scale, ranging from −2 (strongly disagree) to +2 (strongly agree) (Figure 7).

To provide a transparent overview of the system’s performance, a detailed breakdown of the domain-level and item-level numerical results, including mean values and standard deviations, is presented in Table 1.

**Table 1:** User evaluation questionnaire.

Questions		Strongly disagree in %	Disagree in %	Neither agree nor disagree in %	Agree in %	Strongly agree in %	Did not answer in %	Mean	Mode	Standard deviation
The operation of the Apple Vision Pro and the software is intuitive.		0.0	0.0	0.0	20.0	80.0	0.0	1.8	2	0.4
The features of the application are well explained or self-explanatory.		0.0	0.0	6.7	20.0	73.3	0.0	1.7	2	0.6
The desired imaging data can be integrated into the headset quickly and easily.		0.0	0.0	0.0	0.0	0.0	100.0	-	-	-
User-Friendliness								1.7	2	
The system is helpful for improving the visualization of complex brain structures and pathologies.		0.0	0.0	0.0	6.7	86.7	6.7	1.9	2	0.3
The system is a valuable addition to current surgical planning and promotes professional discussion.		0.0	0.0	0.0	33.3	66.7	0.0	1.7	2	0.5
The anatomical structures and surgical approaches can be effectively visualized using the drawing tool.		0.0	0.0	13.3	20.0	66.7	0.0	1.5	2	0.7
Surgical procedures are easier to understand through the 3D visualization provided by the headset and represent an improvement over conventional multiplanar visualization.		0.0	0.0	0.0	6.7	93.3	0.0	1.9	2	0.3
The available features for object visualization and surgical simulation are well organized and provide a clear overview without causing information overload.		0.0	0.0	0.0	46.7	46.7	6.7	1.5	2	0.5
The ability to view the surgical field from any perspective and to virtually position the patient represents a significant advantage for surgical simulation and strategy planning.		0.0	0.0	0.0	13.3	80.0	6.7	1.9	2	0.4
Which software features were particularly helpful and represent a unique advantage compared to other technologies?	Fingernavigation	0.0	0.0	0.0	13.3	66.7	20.0	1.8	2	0.4
	Transparent viewing	0.0	6.7	6.7	20.0	40.0	26.7	1.3	2	1.0
	Selective display of segmented structures	0.0	6.7	0.0	26.7	33.3	33.3	1.3	2	0.9
	Drawing	0.0	0.0	13.3	0.0	73.3	13.3	1.7	2	0.8
The application can also be used for medical student education and training.		0.0	0.0	6.7	0.0	93.3	0.0	1.9	2	0.5
Surgical Planning Utility								1.7	2	
The system is suitable for daily use during morning case conferences.		0.0	0.0	6.7	40.0	53.3	0.0	1.5	2	0.6
There were no technical issues during the use of the system.		0.0	0.0	6.7	13.3	80.0	0.0	1.7	2	0.6
(If technical issues occurred) The technical issues could be resolved independently and quickly.		0.0	0.0	0.0	0.0	0.0	100.0	-	-	-
System Stability								1.6	2	
The visual representation of the anatomical structures is of high quality in terms of resolution, sharpness, and contrast.		0.0	0.0	0.0	6.7	93.3	0.0	1.9	2	0.3
Visualization Quality								1.9	2	
Sum								1.7	2	

### Qualitative findings

The qualitative feedback from structured discussions strongly supported the quantitative data:

- **User-Friendliness:** Surgeons rated the intuitive operation and controller-free interaction gestures exceptionally high, consistently describing the eye- and hand-tracking implementation as a “seamless” transition from physical controllers.
- **Surgical Planning Utility:** The application successfully facilitated real-time manipulation of complex anatomical models. Surgeons noted a significantly improved understanding of surgical strategies, particularly when utilizing transparencies to visualize underlying critical structures (supported by a domain mean score of 1.65).
- **Visualization Quality:** The resolution, sharpness, and contrast of the Apple Vision Pro were highlighted as superior to legacy 3D perception tools, providing a distinct advantage for recognizing micro-anatomical details (supported by a domain mean score of 1.90).
- **System Stability:** The *nextViewer* app demonstrated high stability during both single-user operation and the synchronized multi-user Collaboration Mode, facilitating effective team discussions without disruptive latency.

Qualitative feedback identified the Drawing as well as the Finger Navigation feature—providing immediate crosshair updates in corresponding coronal, sagittal, and axial planes—as the unique selling point (USP) of the software, as it directly translates physical movement into radiological orientation, corroborating the high quantitative scores for spatial orientation.

## Discussion

The Apple Vision Pro spatial computing glasses offer a completely new way of visualizing and interacting with 3D models for planning surgical procedures. The advantages lie particularly in improved spatial perception of complex anatomical structures and in surgical planning through simulation of different surgical approaches. The nextViewer app also improves communication with colleagues during morning meetings to discuss the surgical schedule.

### Descriptive statistics and qualitative feedback

Beyond the numerical aggregate, the qualitative descriptive analysis provided key insights into the system’s unique value proposition:

1. Technical specifications and functional capabilities.

The system leverages specific hardware and software architectures that define its performance:

- **Intuitive Interaction:** Unlike predecessor systems (e.g., Brainlab, Oculus, or HoloLens 2), the Apple Vision Pro's eye and hand-tracking system provides precise, smoother, and faster interaction without the need for additional physical controllers (17, 18, 30–34). Control is achieved solely with eyes and finger gestures.
- **Real-time Navigation Architecture:** Finger navigation provides real-time updates of coronal, sagittal, and axial cross-sectional planes, linked directly to the fingertip's spatial position.
- **Collaborative Infrastructure:** The integration of the collaboration mode allows for synchronized discussion of patient-specific cases either in-person or remotely.

2. Subjective Clinical Impressions

These results reflect the professional qualitative feedback provided by the surgeons:

- **User-Friendliness:** Surgeons reported the interface to be highly intuitive, characterizing the operation as seamless compared to their previous experiences with systems like the Microsoft HoloLens 2.
- **Visualization Utility:** The visual representation of the structures, along with excellent resolution, sharpness, and contrast, were highlighted as major advantages. The ability to utilize transparencies was specifically praised, as it makes it possible to see through a structure to better recognize the areas behind it. Studies from 2025 show that such improved anatomical visualization has the potential to improve patient outcomes (35, 36).
- **Orientation and USP:** The finger navigation feature was widely regarded by the surgeons as the most helpful tool for orientation, serving as a standout element (unique selling point) of the user experience.

### Feature-specific discussion

#### Viewing

The visual representation of the anatomical structures, along with the excellent resolution, sharpness, and contrast of the Apple Vision Pro, were highlighted as major clinical advantages, which is directly supported by our high Likert scores in Visualization Quality. The intuitive viewing mechanics allow for fine-grained scaling and rotation without breaking immersion. The ability to utilize transparencies was specifically praised, as it makes it possible to seamlessly see through a structure to better recognize the critical areas behind it.

#### Drawing

Various trajectories can be drawn into the virtual patient-specific model using finger gestures to optimize preoperative planning. Currently, these trajectories are visualized using a sequence of discrete cubes. In the future, this method will be refined by replacing these discrete geometric elements with continuous mathematical curves, such as splines. This transition will not only eliminate visual kinks to create seamless structures, but it will also drastically reduce the overall polygon count and the number of GPU draw calls required. In untethered spatial computing environments like the Apple Vision Pro, minimizing such rendering complexity directly reduces computational overhead, which in turn mitigates thermal output and significantly extends battery life (37). Additionally, to prevent unintentional marking, future versions will implement a bounding box (region of interest) that restricts drawing capabilities exclusively to the area immediately surrounding the fingertip.

#### Navigation

The standard format for medical image data, DICOM, cannot currently be interpreted natively using the Swift programming language. Therefore, data conversion from DICOM to the PNG image format is necessary to visualize the CT/MRI cross-sectional images. Further conversion to the JSON format enables the orientation data to be used to accurately map coordinates in the corresponding cross-sectional images. To bypass this conversion in the future, the Apple programming library *Metal* must be utilized for direct GPU communication. Clinically, this finger navigation aligns directly with the trajectory outlined by Van Doormaal et al., who emphasize that the integration of augmented reality is crucial for enhancing real-time spatial awareness. While Van Doormaal and colleagues describe the theoretical potential for AR-supported navigation, the nextViewer app actualizes this concept. By instantly translating physical finger movements into multiplanar radiological updates, the system provides the immediate, low-latency visual feedback loop identified as essential for next-generation navigation (14). Furthermore, as Hayeem et al. noted, such real-time integration significantly reduces the cognitive load on the surgeon (38).

#### Collaboration mode

With nextViewer's SharePlay feature, the app provides a platform for exchange directly on the virtual patient model from any location via a FaceTime call. This synchronization represents a critical first step toward telemedicine, opening up unprecedented possibilities. In the future, the app will support identical spatial alignment for users in the same room, making it appear as if all participants are interacting with the exact same physical hologram, while still retaining individual application settings.

#### Areas for optimization and future perspectives

While the overall evaluation score was strong (+1.7), participants suggested essential technical refinements. These include enabling the drawing tool to project lines directly onto the surfaces of virtual anatomical structures, and optimizing the data transfer workflow to decrease latency between planning computers and the Apple Vision Pro. Furthermore, surgeons noted the system's high potential utility for medical student training. However, significant limitations exist regarding integration into existing clinical workflows and the steep learning curve for surgeons without prior spatial computing experience (2). Standardized educational courses will be vital. Finally, widespread adoption remains contingent upon overcoming regulatory hurdles, as many systems still await FDA and MDR certification for active clinical use (28).

A primary limitation of this preliminary clinical utility study is the lack of a controlled, comparative evaluation. Consequently, the findings should not be interpreted as evidence of superiority over existing, established preoperative planning tools. While the subjective quantitative and qualitative feedback is highly promising and highlights significant workflow benefits, future randomized trials are necessary to directly compare the nextViewer app against standard MRI/CT planning workflows or existing VR planning tools. Such comparative studies will be essential to objectively quantify improvements in operative times, cognitive load reduction, and overall patient outcomes.

Furthermore, significant practical limitations exist regarding integration into existing clinical workflows and the steep learning curve for surgeons without prior spatial computing experience. Standardized educational courses will be vital. Finally, widespread adoption remains contingent upon overcoming regulatory hurdles, as many systems still await FDA and MDR certification for active clinical use.

## Conclusions

The developed software for the spatial computing data glasses Apple Vision Pro showed that the integration and use of spatial computing helps to interact with patient-specific virtual models for surgical planning and discussion with our surgeons. As the new hardware was released just one year ago, we see a great future potential spatial computing applications in healthcare.

It is assumed that SC, MR, AR and VR will become standard tools in neurosurgical planning and training in the future and will permanently change the way neurosurgeons plan, simulate, and operate (2).

The future of spatial computing in neurosurgery is promising. In the short term, further development of data glasses will improve ergonomics and image processing speed. In the medium to long term, AI-supported systems that analyze intraoperative data in real time could optimize decision-making, for example in tumor detection or vascular navigation.

## Data Availability

The raw data supporting the conclusions of this article will be made available by the authors, without undue reservation.
