# Supplementary material for: Next viewer–development of a software for the spatial computing data glasses apple vision pro for patient-specific preoperative planning and navigation
Source: Front Digit Health. 2026 Aug 28;8:1764807. doi: 10.3389/fdgth.2026.1764807 (PMC13562247; doi:10.3389/fdgth.2026.1764807)
Supplement: Supplementary file 1 [file Datasheet1.pdf]

Questions		Strongly disagree in %	disagree in %	Neither Agree nor disagree in %	Agree in %	Strongly agree in %	Did not answer in %	Mean	Mode	Standard Deviation
The operation of the Apple Vision Pro and the software is intuitive.		0,0	0,0	0,0	20,0	80,0	0,0	1,8	2	0,4
The features of the application are well explained or self-explanatory.		0,0	0,0	6,7	20,0	73,3	0,0	1,7	2	0,6
**User-Friendliness**								1,7	2	
The system is helpful for improving the visualization of complex brain structures and pathologies.		0,0	0,0	0,0	6,7	86,7	6,7	1,9	2	0,3
The system is a valuable addition to current surgical planning and promotes professional discussion.		0,0	0,0	0,0	33,3	66,7	0,0	1,7	2	0,5
The anatomical structures and surgical approaches can be effectively visualized using the drawing tool.		0,0	0,0	13,3	20,0	66,7	0,0	1,5	2	0,7
Surgical procedures are easier to understand through the 3D visualization provided by the headset and represent an improvement over conventional multiplanar visualization.		0,0	0,0	0,0	6,7	93,3	0,0	1,9	2	0,3
The available features for object visualization and surgical simulation are well organized and provide a clear overview without causing information overload.		0,0	0,0	0,0	46,7	46,7	6,7	1,5	2	0,5
The ability to view the surgical field from any perspective and to virtually position the patient represents a significant advantage for surgical simulation and strategy planning.		0,0	0,0	0,0	13,3	80,0	6,7	1,9	2	0,4
Which software features were particularly helpful and represent a unique advantage compared to other technologies?	Fingernavigation	0,0	0,0	0,0	13,3	66,7	20,0	1,8	2	0,4
	Transparent Viewing	0,0	6,7	6,7	20,0	40,0	26,7	1,3	2	1,0
	Selective display of segmented structures	0,0	6,7	0,0	26,7	33,3	33,3	1,3	2	0,9
	Drawing	0,0	0,0	13,3	0,0	73,3	13,3	1,7	2	0,8
The application can also be used for medical student education and training.		0,0	0,0	6,7	0,0	93,3	0,0	1,9	2	0,5
**Surgical Planning Utility**								1,7	2	
The system is suitable for daily use during morning case conferences.		0,0	0,0	6,7	40,0	53,3	0,0	1,5	2	0,6
There were no technical issues during the use of the system.		0,0	0,0	6,7	13,3	80,0	0,0	1,7	2	0,6
(If technical issues occurred) The technical issues could be resolved independently and quickly.		0,0	0,0	0,0	0,0	0,0	100,0	-	-	-
**System Stability**								1,6	2	
The visual representation of the anatomical structures is of high quality in terms of resolution, sharpness, and contrast.		0,0	0,0	0,0	6,7	93,3	0,0	1,9	2	0,3
**Visualization Quality**								1,9	2	
Sum								1,7	2	
